# Adsorption and molecular siting of CO_2_, water, and other gases in the superhydrophobic, flexible pores of FMOF-1 from experiment and simulation†
†Electronic supplementary information (ESI) available. See DOI: 10.1039/c7sc00278e



**DOI:** 10.1039/c7sc00278e

**Published:** 2017-03-10

**Authors:** Peyman Z. Moghadam, Joshua F. Ivy, Ravi K. Arvapally, Antonio M. dos Santos, John C. Pearson, Li Zhang, Emmanouil Tylianakis, Pritha Ghosh, Iain W. H. Oswald, Ushasree Kaipa, Xiaoping Wang, Angela K. Wilson, Randall Q. Snurr, Mohammad A. Omary

**Affiliations:** a Department of Chemical & Biological Engineering , Northwestern University , 2145 Sheridan Road , Evanston , IL 60208-3120 , USA . Email: snurr@northwestern.edu; b Department of Chemistry , University of North Texas , Denton , Texas 76203 , USA . Email: wilson@chemistry.msu.edu ; Email: omary@unt.edu; c Neutron Sciences Directorate , Oak Ridge National Laboratory , Oak Ridge , TN 37831 , USA . Email: wangx@ornl.gov; d Department of Chemistry , Zhejiang Sci-Tech University , Hangzhou , China; e Department of Materials Science & Technology , University of Crete , Voutes Campus , Heraklion , Crete GR-71003 , Greece; f Department of Chemistry , Michigan State University , East Lansing , MI 48824-1322 , USA

## Abstract

FMOF-1 is a flexible, superhydrophobic metal–organic framework with a network of channels and side pockets decorated with –CF_3_ groups. CO_2_ adsorption isotherms measured between 278 and 313 K and up to 55 bar reveal a maximum uptake of *ca.* 6.16 mol kg^–1^ (11.0 mol L^–1^) and unusual isotherm shapes at the higher temperatures, suggesting framework expansion. We used neutron diffraction and molecular simulations to investigate the framework expansion behaviour and the accessibility of the small pockets to N_2_, O_2_, and CO_2_. Neutron diffraction *in situ* experiments on the crystalline powder show that CO_2_ molecules are favourably adsorbed at three distinct adsorption sites in the large channels of FMOF-1 and cannot access the small pockets in FMOF-1 at 290 K and oversaturated pressure at 61 bar. Stepped adsorption isotherms for N_2_ and O_2_ at 77 K can be explained by combining Monte Carlo simulations in several different crystal structures of FMOF-1 obtained from neutron and X-ray diffraction under different conditions. A similar analysis is successful for CO_2_ adsorption at 278 and 283 K up to *ca.* 30 bar; however, at 298 K and pressures above 30 bar, the results suggest even more substantial expansion of the FMOF-1 framework. The measured contact angle for water on an FMOF-1 pellet is 158°, demonstrating superhydrophobicity. Simulations and adsorption measurements also show that FMOF-1 is hydrophobic and water is not adsorbed in FMOF-1 at room temperature. Simulated mixture isotherms of CO_2_ in the presence of 80% relative humidity predict that water does not influence the CO_2_ adsorption in FMOF-1, suggesting that hydrophobic MOFs could hold promise for CO_2_ capture from humid gas streams.

## Abstract 2

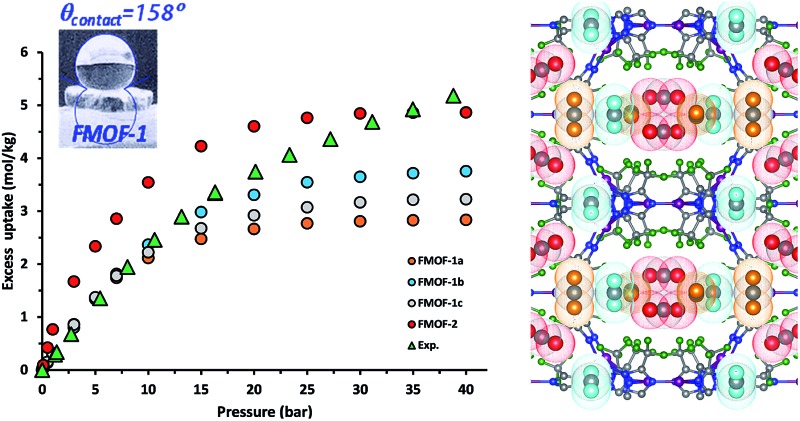
CO_2_ isotherms for FMOF-1 reveal 11.0 mol L^–1^ max uptake and suggest framework expansion, substantiated by *in situ* neutron diffraction and GCMC simulations.

## Introduction

Capturing carbon from flue gas is an important challenge, as fossil fuel combustion continues to be a primary source of energy.^1,2^ The search for adsorbents capable of capturing large amounts of CO_2_ has led to many studies of adsorption in nanoporous materials known as metal–organic frameworks (MOFs).^3–5^ MOFs have been shown to be promising for a number of separation applications including CO_2_ capture at the low partial pressures relevant to flue gas.^6–11^ In an early example, Yazaydin *et al.* screened 14 MOFs and concluded that the M/DOBDC series shows exceptionally high CO_2_ capacity at room temperature.^12^ This can be attributed to the high density of open metal sites, and other studies on CO_2_ capture in MOFs have shown similar results.^13^ After the CO_2_ is captured, it must be stored or used afterward. One option is permanent subterranean storage as a pressurized liquid. Alternatively, it could be converted and used as other chemical products. Darensbourg *et al.* recently reported CO_2_ capture in a MOF, HKUST-1, to perform a copolymerization with propylene oxide with a 49.9% conversion rate.^2^

When assessing adsorbents for use with flue gas, the presence of water vapor cannot be ignored.^9^ For example, it has been shown that M/DOBDC MOFs show a significant decrease in CO_2_ capture capacity under humid conditions.^14,15^ Hydrophobic MOFs could be an attractive alternative, due to their ability to withstand humid conditions and suppress competitive adsorption of water. A number of studies in the literature have investigated the effects of fluorination and hydrophobicity in MOFs.^16–22^ FMOF-1 is a fluorous metal–organic framework first synthesized by Yang *et al.* ^20^ It is formed by the reaction of a perfluorinated ligand (3,5-bis(trifluoromethyl)-1,2,4-triazolate (Tz^–^)) with a Ag^+^ precursor, leading to {Ag_2_[Ag_4_Tz_6_]}. FMOF-1 exhibits a perfluorinated structure, and the many CF_3_ groups lining its channels and small pockets imbue it with hydrophobicity.^20^ In principle, the CO_2_ quadrupole should be able to interact with the polar C–F groups in FMOF-1, but CO_2_ adsorption measurements have not been reported for FMOF-1. In addition, while FMOF-1 has been shown to experience enormous breathing behaviour as a function of temperature either under vacuum or in the presence of N_2_,^23^ the evolution of the FMOF-1 structure has not been studied previously in the presence of other guest molecules or at elevated pressures. Given that changes in framework structure can have a significant effect on the adsorption properties of a porous material, we investigate in this work whether framework flexibility is a general feature of FMOF-1 and the effect of flexibility on adsorption uptake by investigating a wide variety of guest molecules, with a particular focus on CO_2_.

In this paper, experimental CO_2_ adsorption isotherms, contact angle measurement of water drops, and *in situ* neutron diffraction results during CO_2_ adsorption are reported. Also reported are grand canonical Monte Carlo and quantum mechanical simulations in FMOF-1 to model the adsorption of CO_2_ and other guest molecules, including adsorption of CO_2_ under humid conditions. The work illustrates the power of a strong feedback loop between experiment and modeling. For example, neutron diffraction studies provided possible crystal structures for simulations of CO_2_ adsorption, and measured isotherms and heats of adsorption provided validation of predictions from modeling. In turn, modeling provided insight about molecular siting in FMOF-1 and predictions about the CO_2_ capture performance under humid conditions. The flexible nature of FMOF-1 was investigated *via* simulation using four different FMOF-1 structures obtained under different experimental conditions. Correlations between the framework structure and guest uptake were established for three classes of guest molecules, including diatomics at one extreme and bulky hydrocarbons at the other, with CO_2_ representing an intermediate category.

## Methods

### FMOF-1 synthesis and adsorption measurements

FMOF-1 was prepared using previously published methods.^20,23^ Nitrogen adsorption isotherms at 77 K were measured with a Micromeritics ASAP 2020. CO_2_ adsorption measurements were carried out by a VTI/TA Gravimetric High Pressure Sorption Analyzer. This VTI/TA system is equipped with ultra-high vacuum and is capable of variable temperature measurements from –196 °C to 1000 °C. It has a flow dosing manifold for high pressure studies and achieves 0.1 μg resolution with a CI Electronics microbalance. Typically, 100 mg of sample was used for adsorption measurements. Before each measurement, the sample was purged with helium then evacuated for 60 minutes at 60 °C. Measurements were performed at 5 degree intervals from 5° to 40 °C and pressures up to 53 bar or the critical pressure of CO_2_ at the set temperature. Isosteric heats of adsorption were derived using a set of isotherms at different temperatures and the Clausius–Clapeyron equation.^24^ High purity CO_2_ gas was used for the adsorption studies.

### Contact angle measurements

Contact angle measurements were done using a Rame-hart manual goniometer (Model # 50-00-1150). Static contact angle was measured. A single drop of water was added using the syringe attached to the goniometer and then the contact angle was measured on the static sessile drop with the gauge provided.

### Neutron powder diffraction measurements

*In situ* neutron powder diffraction measurements were performed at the SNAP beamline of the Spallation Neutron Source (SNS) at Oak Ridge National Laboratory (ORNL). SNAP is a high flux and medium resolution time-of-flight diffractometer, with tunable detector placement and incident energy range. For this experiment, measurements were made with the two detector banks placed at 90 and 48 degrees, and the wavelength band used was 3.5 Å wide and centered at 6.4 Å. This configuration enabled sampling of Bragg reflections in the range 3–17 Å, enough to sample the longest (in *d*-spacing) reflections of the sample. Fully activated FMOF-1 powder sample was loaded into a gas cell fabricated with a null scattering TiZr alloy (1 : 2.08 Zr : Ti molar ratio) and warmed to 320 K in a dynamic vacuum. The gas cell was then cooled *via* 200 mbar of He exchange gas on a top loading cold cycle refrigerator cryostat to 290 K for data collection on the bare FMOF-1. CO_2_ was then loaded slowly into the FMOF-1 sample at 290 K using a computer-controlled automated gas handling system and held for an hour at 61 bar to ensure the sample cell maintained over the CO_2_ saturation pressure (53.2 bar) before and during data collection. High purity CO_2_ gas stored at room temperature (296 K) was used directly from the cylinder with no further purification.

Neutron diffraction data were analyzed using the GSAS II package.^25^ The locations of CO_2_ molecules in FMOF-1 after CO_2_ loading were obtained from difference Fourier map and refined accordingly with distance constraints.

### Simulation details

Grand canonical Monte Carlo (GCMC) simulations were employed to investigate the adsorption of N_2_, O_2_, CO_2_, H_2_O, *n*-hexane, and benzene. For each pressure point of the isotherm, 1 × 10^5^ GCMC cycles were used for equilibration, after which another 1 × 10^5^ cycles were used to calculate the average properties. For water simulations, we used 5 × 10^5^ cycles each for equilibration and production runs. Each GCMC cycle is made up of *N* steps, where *N* is the number of adsorbates in the simulation box. (The number of steps per cycle is not allowed to be lower than 20; so if there are fewer than 20 adsorbates in the simulation box, a cycle consists of 20 steps.) The TraPPE force field was used to model all adsorbates (N_2_,^26^ O_2_,^27^ CO_2_,^26^ *n*-hexane,^28^ and benzene^29^) except for water, which was described with the TIP4P model.^30^ Lennard-Jones parameters for the framework atoms were taken from the Universal Force Field.^31^ Cross Lennard-Jones parameters were determined by Lorentz–Berthelot mixing rules. The partial charges for CF_3_ groups were adopted from the work of Dalvi *et al.*,^32^ while partial charges for the rest of the framework were obtained from density functional theory calculations at the B3LYP level of theory using the ChelpG method.^33^ Lennard-Jones parameters and partial atomic charges for the adsorbates and FMOF-1 are all listed in the ESI (Tables S3 and S4†). A cutoff distance of 12.8 Å was used for all Lennard-Jones interactions, and tail corrections were neglected. Long-range electrostatic interactions were accounted for using the Ewald summation method. The simulation box was constructed of 4 (2 × 2 × 1) unit cells with periodic boundary conditions applied in all directions.

Framework atoms were held fixed during the GCMC simulations. To explore the effect of framework flexibility on the adsorption properties, we performed simulations on three different FMOF-1 crystal structures along with the related FMOF-2 polymorph (see Fig. S12 in the ESI†) for CO_2_ adsorption at room temperature. The first published crystal structure of FMOF-1 was obtained under vacuum at 100 K,^23^ and this structure shall be referred to as FMOF-1a in this work. A structure obtained under a nitrogen stream at 90 K (ref. 23) shall be referred to here as FMOF-1b. The third FMOF-1 structure is a heretofore-unpublished structure obtained under a carbon dioxide stream at 61 bar and 290 K and will be referred to as FMOF-1c.

### Quantum chemical methods

Density functional theory (DFT) was used to determine the binding free energy of molecules at their most likely adsorption sites. The binding sites in the cylindrical channel and small cavity were simulated individually. The cylindrical channel was truncated from the FMOF-1a structure and was composed of 11 Tz ligands and 9 Ag atoms. The small pocket was truncated from the FMOF-1b structure containing 14 Tz ligands and 10 Ag atoms. Both sites fully accounted for the correct coordination geometry of all metal centers using the neutral singlet state. Equilibrium geometries for guest molecules were found, holding the framework static. The NWCHEM software package was used for all quantum chemical calculations.^34^ We chose to use the BPE0 functional with 6-311G* Pople basis sets for non-metal atoms and the Stuttgart-97 Effective Core Potential and respective basis sets for silver.^35–38^ The empirical dispersion correction DFT-D3 was added to address the long-range effects.^39^ The inclusion of dispersion corrections with DFT has been shown to be necessary for the prediction of MOF structures, creating force field parameters, predicting changes in structure, and determining water adsorption sites.^40–44^ Orbital comparisons were made using the Mulliken population analysis. The total population density of the empty *versus* the occupied site used the same basis set and framework coordinates.

## Results and discussion

### CO_2_ adsorption and contact angle studies

**Fig. 1:**
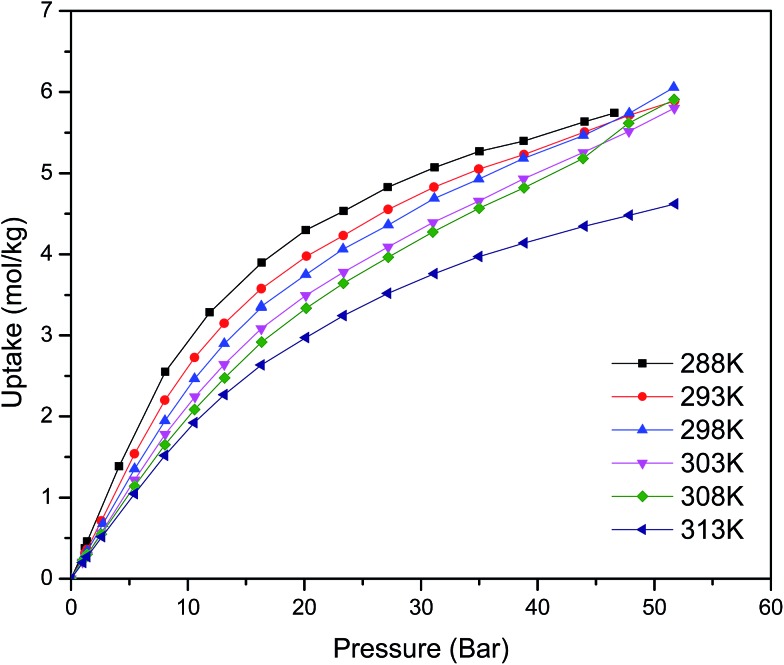
CO_2_ excess adsorption isotherms of FMOF-1 at various near-ambient temperatures.

Fig. 1 shows the CO_2_ adsorption isotherms for FMOF-1 at near-ambient temperatures up to 55 bar. The maximum uptake experimentally measured is *ca.* 6.16 mol kg^–1^ (11.0 mol L^–1^; 27.1 wt%; 483 kg m^–3^; 248 V STP V^–1^) at 298 K and 55 bar. This uptake is more than 2 times higher than that predicted^45^ in 2008 based on the rigid structure of FMOF-1 reported in 2007,^20^ necessitating further modeling that takes into account the framework flexibility (*vide infra*). The isotherms do not reach a plateau and are inconsistent with a type I adsorption behaviour over this temperature and pressure range, again suggesting flexibility of the framework. This behaviour was determined not to be due to a systematic error in the experimental data, as it has been reproduced multiple times; see Fig. S2 (ESI†). Up to 30 bar, the isotherms can be fit very well with a Toth isotherm (Fig. S1†), and above this pressure the isotherms continue to rise, with a visible inflection at some temperatures.

**Fig. 2:**
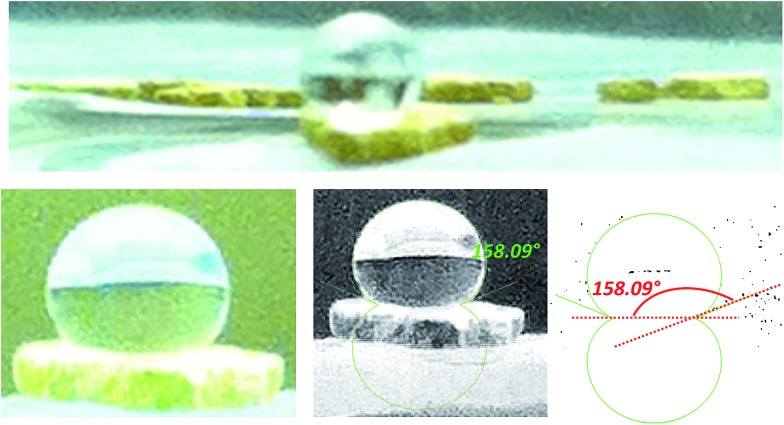
Images of a water drop on a pellet of FMOF-1 that formed after the high-pressure CO_2_ adsorption isotherm experiment shown in Fig. 1; most water droplets in the attempted contact angle experiments deflected off the surface of the pellet quickly as complete spheres. The bottom-right and bottom-middle images show the processing of the bottom-left raw image using the LBADSA plugin for the ImageJ software.^51^

Upon removing the sample from the instrument after the CO_2_ adsorption studies, we noticed that the powder sample had transformed into a flat, yellow-coloured pellet. TGA and IR studies for pieces of such a pellet revealed essentially identical profiles to the powder sample of FMOF-1. We performed a contact angle experiment for a water droplet upon this pellet. This process proved difficult, as the water droplets tended to bounce and deflect off of the surface of the pellet rather quickly and as complete spheres, suggesting a rather extreme superhydrophobic behaviour of the material. Processing of the resulting image yielded a contact angle of ∼158, as shown in Fig. 2, clearly indicating a superhydrophobic behaviour and consistent with the insignificant water adsorption reported earlier for FMOF-1.^46–48^ The work herein, therefore, ascertains that FMOF-1 belongs to the “superhydrophobic” category of MOFs, similar to very few other frameworks verified as such from contact angle measurements.^22,49,50^

### Neutron diffraction studies

**Fig. 3:**
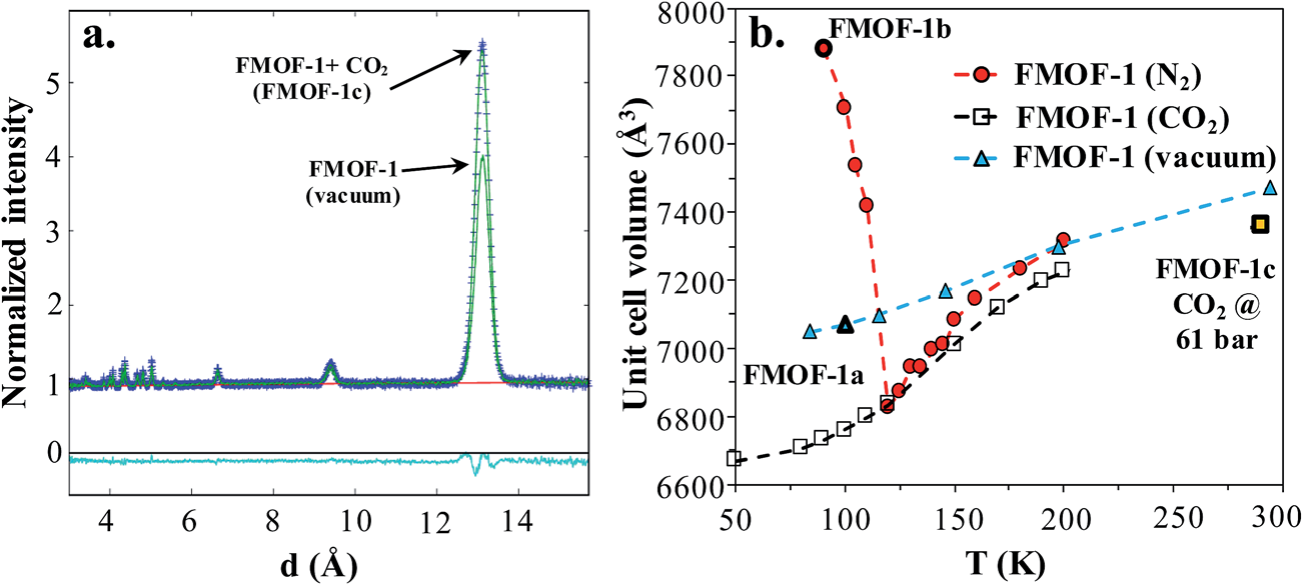
(a) FMOF-1 neutron powder diffraction patterns measured at 290 K in vacuum (solid green line) and under 61 bar of CO_2_ (blue crosses). The residuals (cyan) underneath the zero line are the difference of the observed and refined neutron diffraction profiles of the FMOF-1 sample with adsorbed CO_2_. w*R* = 1.606%, GOF = 1.87, *N*_obs_ = 1658, *N*_vals_ = 14. Space group *I*42*d*, *a* = 13.9713(7) Å, *c* = 37.713(4) Å, *V* = 7361.4(7) Å^3^. (b) Temperature dependence of unit cell volume of FMOF-1 under vacuum,^23^ constant stream of N_2_ at atmospheric pressure^23^ and CO_2_ at 4.8 bar. The unit cell volume of FMOF-1 under 61 bar and 290 K (FMOF-1c) is also shown for comparison.

Neutron powder diffraction measurements were performed at the ORNL Spallation Neutrons and Pressure Diffractometer (SNAP) (Table S1†). Fig. 3a compares the neutron powder diffraction patterns for the evacuated FMOF-1 and the structure under 61 bar of CO_2_ at 290 K. Under this oversaturated pressure condition, the free CO_2_ molecules stay in the liquid state. The increase in intensity for the 011 peak at ∼13 Å after *in situ* CO_2_ loading is clearly discernible. The FMOF-1 sample with adsorbed CO_2_ was cooled to 230 K and the pressure was reduced to 4.8 bar for low-temperature measurements. The pressure in the TiZr cell was controlled *via* a computer-controlled gas handling system. Fig. S4 (ESI†) shows the evolution of the neutron powder diffraction patterns of the FMOF-1 sample with adsorbed CO_2_. No evidence of solid CO_2_ was observed in the structural refinement of the CO_2_ loaded samples, indicating that all CO_2_ was adsorbed into FMOF-1.

Fig. 3b shows the temperature dependence of the unit cell volume of FMOF-1 at a static pressure of 4.8 bar of CO_2_ loading and compares it with the results obtained under N_2_ stream^23^ and vacuum.^23^ The smooth change in the unit cell volume in CO_2_ (open squares) is in contrast to that of FMOF-1 with adsorbed N_2_ (red circles in Fig. 3b), in which the loading of N_2_ molecules into small cavities of FMOF-1 at temperatures below 119 K causes a huge negative thermal expansion in the crystal structure.^23^

**Fig. 4:**
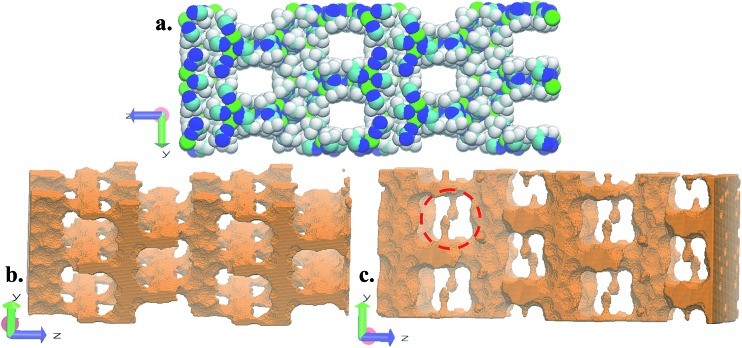
Schematic views of FMOF-1 channels. (a) Front view of FMOF-1 framework. C, cyan; F, white; N, blue and Ag, green. (b) Illustration of cylindrical channel voids using a 4 Å probe. The cylindrical channels extend along the *x*- and *y*-directions. (c) Representation of channel voids and small pockets protruding alongside the channels highlighted in red circles using a 1 Å probe. Views in (b) and (c) are calculated using the method described by Sarkisov and Harrison.^52^

FMOF-1 has two types of pores. The first type are large cylindrical channels extending along both the *x*- and *y*-directions (Fig. 4a and b), with CF_3_ groups protruding into the channels. The second pore type is a small cavity, and two pairs of CF_3_ groups function as a gate between the small cavity and the large cylindrical channel (see the red circle in Fig. 4c). The pore size distribution and full geometric characterization of FMOF-1 can be found in Fig. S5 and Table S2.†

**Fig. 5:**
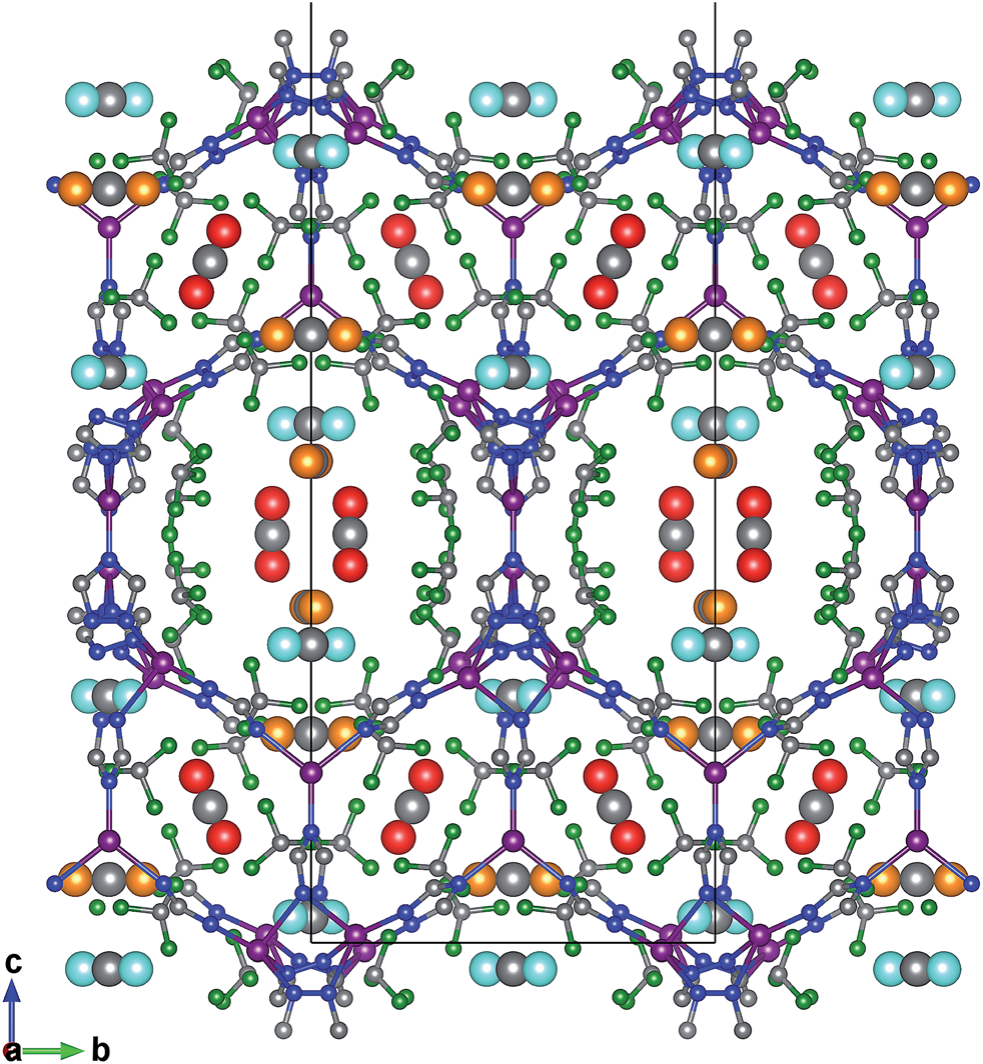
Crystal structure of adsorbed CO_2_ from neutron powder diffraction at 290 K and 61 bar, viewed along the crystallographic *a*-axis. The three CO_2_ adsorption sites in FMOF-1 are shown as site I near CF_3_ groups at corners with O atoms depicted as cyan spheres; site II near CF_3_ groups along *c* direction with O atoms depicted as red spheres; and site III in the direction of the large channels with O atoms depicted as orange spheres.

The favourable adsorption sites for CO_2_ molecules in FMOF-1 were obtained from difference Fourier map and with the CO_2_ molecules refined with distance constraints. Difference Fourier map from initial Rietveld refinements indicate that the CO_2_ molecules are located only in the large channels of FMOF-1 at three unique sites. The FMOF-1 structure loaded with CO_2_ molecules at 61 bar and 290 K is shown in Fig. 5. The oxygen atoms in the three primary CO_2_ adsorption sites are shown as cyan spheres at site I near the framework –CF_3_ groups at corners; as red spheres at site II near –CF_3_ groups along the crystallographic *c* direction; and as orange spheres at site III in the direction of the large channels. Since there is no CO_2_ in the small cavities, when all three CO_2_ adsorption sites are fully occupied, there are 24 CO_2_ molecules in the unit cell (3.3 mol kg^–1^). This limits the CO_2_ uptake capacity in the large cavity to 6 CO_2_ molecules per {Ag_2_[Ag_4_Tz_6_] repeat unit.

### Molecular simulation studies and connection to experimental data

**Fig. 6:**
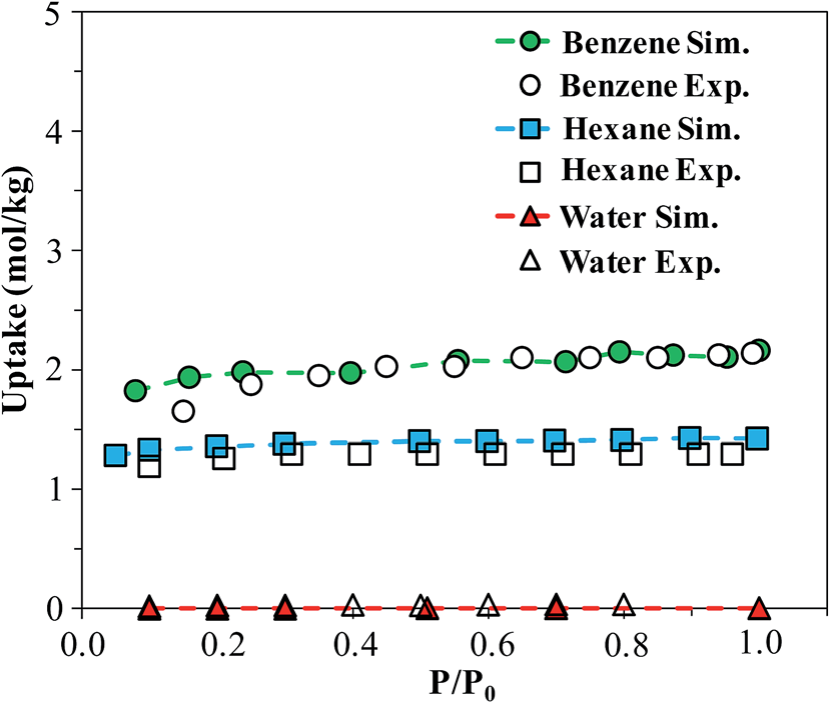
Simulated and experimental adsorption isotherms for benzene, *n*-hexane, and water in FMOF-1a at 298 K. *P*_0_ is the experimental saturation pressure of each adsorbate.

In order to verify our simulation parameters, we began by reproducing previously published experimental isotherms for benzene, *n*-hexane, and water in FMOF-1. Fig. 6 compares simulated results in FMOF-1a to the experimental isotherms. We see a very good agreement between simulation and experiment for benzene and hexane through the entire pressure range, with saturation loadings around 2 mol kg^–1^ and 1.2 mol kg^–1^, respectively. Additionally, both experiment and simulation show no appreciable adsorption of water, thereby confirming the hydrophobic nature of FMOF-1.

**Fig. 7:**
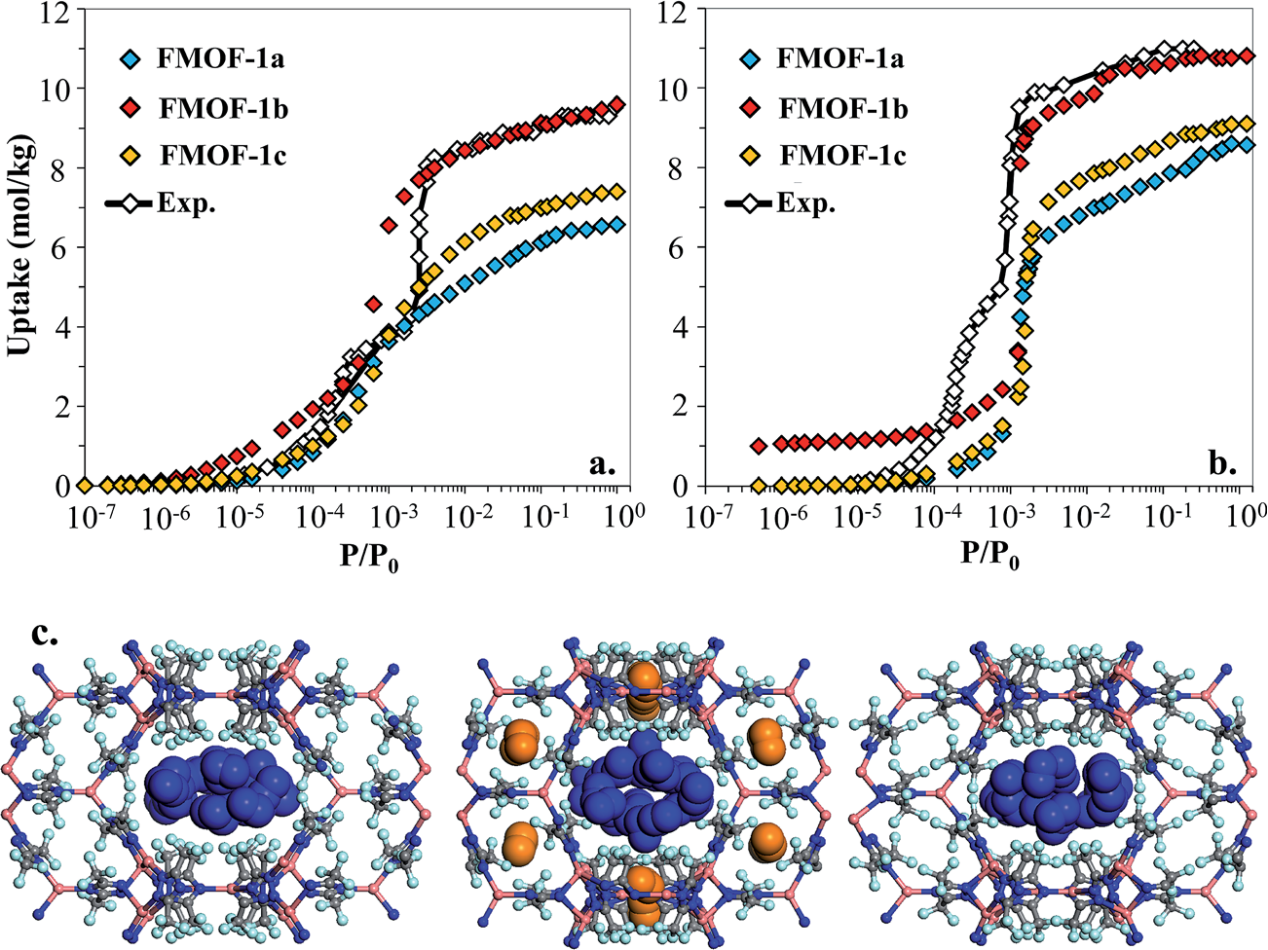
Experimental adsorption isotherms for (a) N_2_ and (b) O_2_ at 77 K compared to simulated isotherms in three FMOF-1 structures. In (c) GCMC simulation snapshots for N_2_ at saturation loading are shown for FMOF-1a (left), FMOF-1b (middle) and FMOF-1c (right) at 77 K. Nitrogen molecules adsorbed in FMOF-1 large channels and small pockets are illustrated with blue and orange vdW representation, respectively. *P*_0_ is the experimental saturation pressure of each adsorbate.

However, as shown in Fig. 7a and b, simulated isotherms of N_2_ and O_2_ adsorption in FMOF-1a drastically underpredict the saturation loading from experiment by almost 3 mol kg^–1^ in both cases. The N_2_ and O_2_ experimental isotherms show two steps, but the FMOF-1a simulated isotherms do not exhibit this behaviour. This pronounced step can be ascribed to the flexibility of the framework upon adsorption, meaning that N_2_ or O_2_ adsorption leads to structural changes of the framework, allowing for more molecules to be adsorbed in the framework. Similar adsorption isotherms have been observed in some ZIFs and the MIL series of MOFs due to framework flexibility.^53–59^ Since the framework is held rigid in our simulations, we approximated the effect of framework flexibility by simulating isotherms with three different FMOF-1 structures, FMOF-1a, FMOF-1b, and FMOF-1c. FMOF-1b has a larger unit cell volume and channel size than FMOF-1a; the largest cavity diameter in FMOF-1a is 6.1 Å, while in FMOF-1b it is 6.8 Å (see Table S2†). The channel size in FMOF-1c lies between FMOF-1a and FMOF-1b, with a 6.3 Å diameter. Recall from Fig. 3 that the unit cell volume of FMOF-1 varies with temperature and CO_2_ loading. Fig. 7 also shows adsorption isotherms of N_2_ and O_2_ in FMOF-1b and FMOF-1c. The two-step adsorption behaviour observed experimentally can be better described when the simulated isotherms of these different structures are considered. At low loadings, the FMOF-1a and FMOF-1c results agree better with experiment, while at high loadings the saturation loading in the expanded structure (*i.e.* FMOF-1b) is closer to experiment. The maximum amounts adsorbed in FMOF-1b for N_2_ and O_2_ are 9.6 and 10.7 mol kg^–1^, respectively, which are in excellent agreement with the experimental values.

The results shown in Fig. 7 corroborate that the framework of FMOF-1 goes through structural expansion upon adsorption of guest molecules. The small cavity in FMOF-1b has a diameter of *ca.* 3.4 Å, which is much larger than the small cavity in FMOF-1a or FMOF-1c (*ca.* 2.5 Å) (Table S2†). The kinetic diameters of N_2_ and O_2_ are 3.64 Å and 3.46 Å,^3^ respectively, which suggests that at most one N_2_ or O_2_ molecule can fit in each small pocket. Interestingly, the window sizes connecting the small pockets to the larger channels are smaller than the kinetic diameters of N_2_ and O_2_. However, kinetic diameters do not account for the orientation of the molecule – the cross section of a N_2_ or O_2_ molecule is *ca.* 2.9 Å,^60^ indicating that only FMOF-1b has a window size large enough to admit an N_2_ or O_2_ molecule. Additionally, since the FMOF-1b structure was obtained at 90 K and the isotherms are measured at 77 K, it is possible that the window size could also be larger at the lower temperature.

To investigate the placement of N_2_ within FMOF-1, we compared the position of N_2_ molecules adsorbed in the FMOF-1 structures at the saturation loading in the GCMC simulations at 77 K (Fig. 7c). In FMOF-1a and FMOF-1c, N_2_ molecules adsorb only in the large cylindrical channels. On the other hand, in FMOF-1b, the N_2_ molecules are adsorbed first in the small pockets at low pressures (Fig. S6†), and it is at the higher pressure that N_2_ molecules adsorb in the larger cylindrical channels. In FMOF-1b, molecules prefer to adsorb in the small pores due to the strong interactions between the adsorbed molecules and the Tz ring pairs, and only as the pressure increases do the large channels fill up. Given that only one N_2_ molecule can occupy each small pocket, the contribution of small pockets to the total amount adsorbed is 1.1 mol kg^–1^ (*i.e.*, 8 molecules per unit cell of FMOF-1b). This uptake accounts for 11% of the total amount adsorbed. The remainder of the adsorption difference between FMOF-1b and FMOF-1c or FMOF-1a is due to additional adsorption in the large channels. As shown in Fig. S6,† a similar adsorption mechanism and placement was observed for O_2_ at 77 K. This finding further confirms that the structural expansion of FMOF-1 occurs not only in the small pockets but also increases the capacity of the larger channels for small adsorbates. It is worth mentioning that the simulated adsorption isotherms of larger adsorbates such as *n*-hexane and benzene in FMOF-1b and FMOF-1c show no significant differences when compared to the isotherm calculated for FMOF-1a (Fig. S7†). Toth fits for the *n*-hexane and benzene adsorption isotherms obtained for FMOF-1 agree very well with both the experimental data and the simulation thereof (Fig. S8a and S8b,† respectively). The kinetic diameters of *n*-hexane and benzene are 4.3 Å and 5.9 Å, respectively, both of which are much larger than the window and pore size of the small pockets. Therefore, the saturation loading of these large molecules is mainly determined by the volume of the cylindrical channels, resulting in a simple type-1 adsorption isotherm profile, with no adsorption in the smaller pores and no consequent steps in the adsorption isotherms. This finding further demonstrates that, while the accessibility of the smaller pockets and larger main channels for different expansion levels of FMOF-1 have significant effects on the adsorption properties of small adsorbates (*e.g.*, N_2_, O_2_ and H_2_), these structural changes have little effect on the uptake of larger adsorbates (*e.g.*, benzene, *n*-hexane, and toluene).

**Fig. 8:**
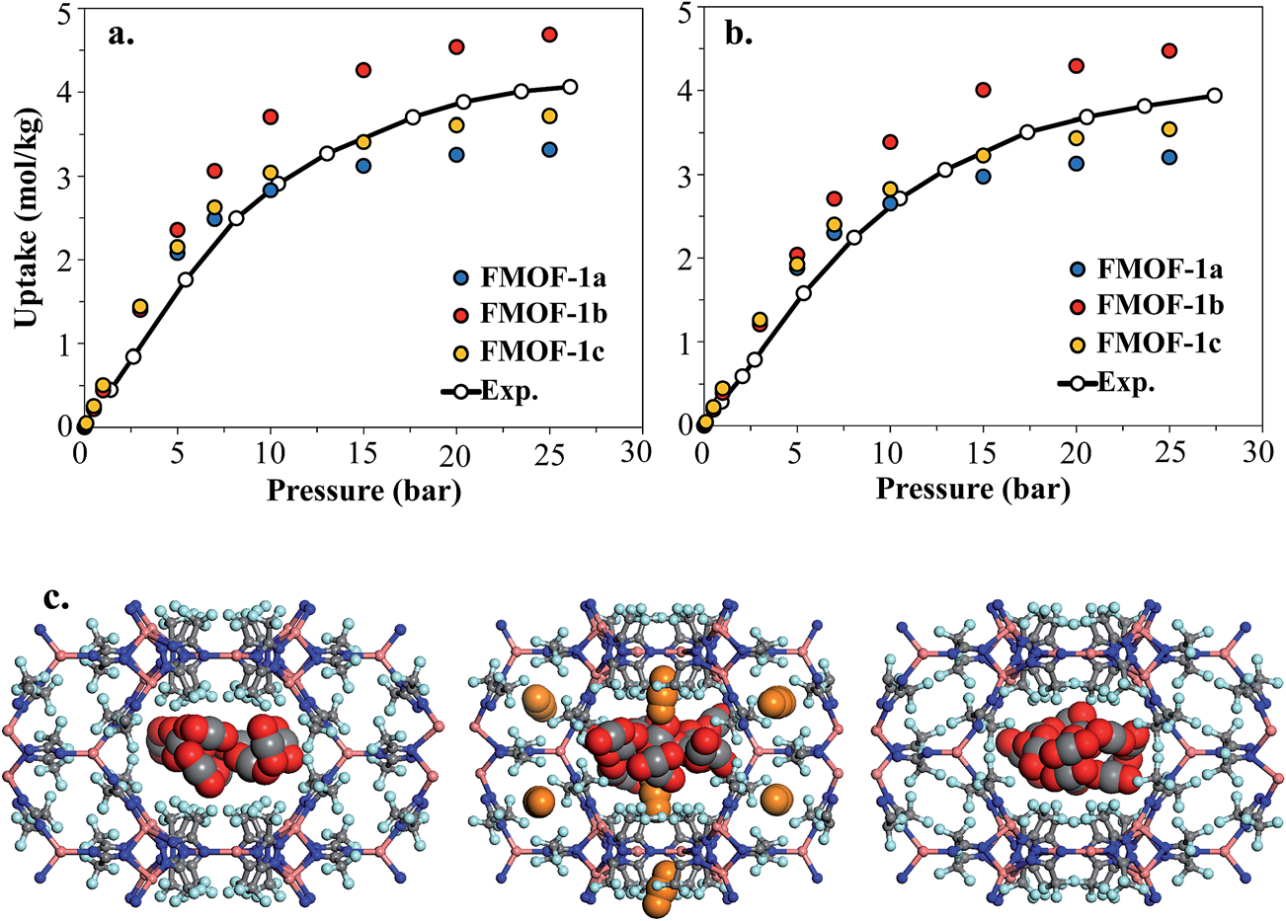
Experimental adsorption isotherms for CO_2_ and simulated isotherms in three FMOF-1 structures at (a) 278 K and (b) 283 K. In (c) GCMC simulation snapshots at saturation loading are shown for FMOF-1a (left), FMOF-1b (middle) and FMOF-1c (right) for CO_2_ at 278 K. CO_2_ molecules are illustrated in vdW representation, with those adsorbed in the large channels in red and grey and those at the entrance to the small pockets in orange. The experimental and simulated results are excess adsorption isotherms.

Fig. 8 shows the experimental CO_2_ adsorption isotherms in FMOF-1 structures at 278 K and 283 K, along with simulated data for comparison. This initial comparison is restricted to 30 bar, where the experimental isotherms can be fit with a Toth isotherm and FMOF-1 shows less pronounced flexibility and expansion. The simulated isotherms in all three structures agree reasonably well with the experimental data at low pressure. The difference between experiment and simulation increases for FMOF-1b as the pressure increases. At saturation loadings, better agreement between experiment and simulation results is observed for FMOF-1c, the structure that was obtained under the stream of CO_2_ (see Fig. 3). The simulation snapshots at high loading show that CO_2_ molecules occupy only the large channels in FMOF-1a and FMOF-1c, since the pockets are too small for CO_2_ (kinetic diameter: 3.4 Å). Interestingly, even in the expanded structure of FMOF-1b, the CO_2_ molecules cannot be fully accommodated inside the small pockets and stay at the windows connecting the large channels to the small pockets as highlighted in the simulation snapshots in Fig. 8c and the CO_2_ density profiles shown at high pressure in Fig. S9.†

**Fig. 9:**
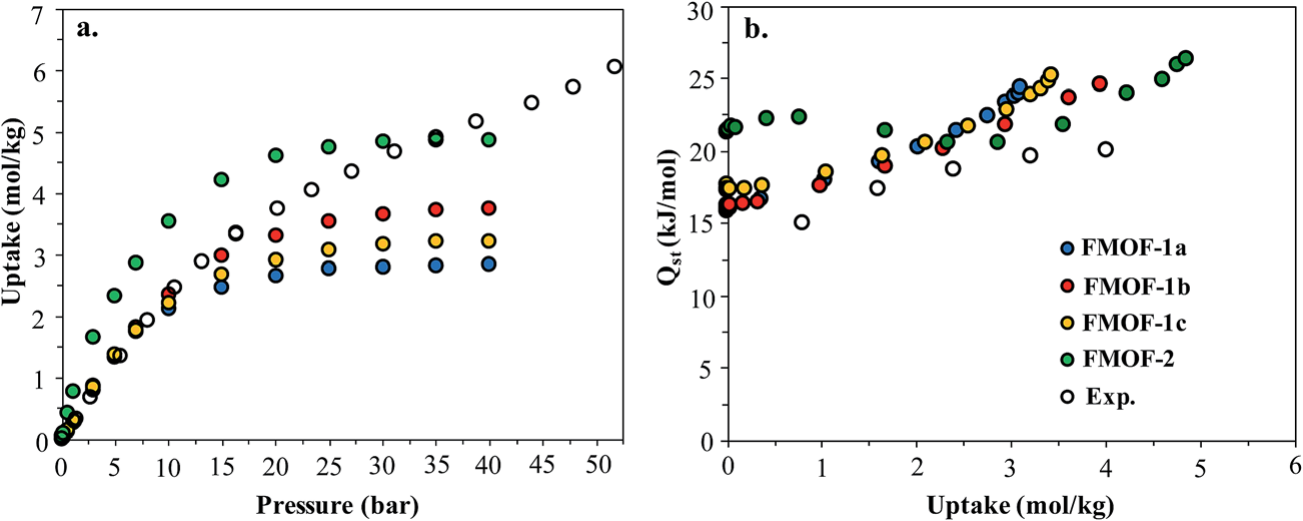
(a) Experimental excess adsorption isotherms for CO_2_ at 298 K in FMOF-1 and simulated excess isotherms in three FMOF-1 structures and FMOF-2. (b) Experimental and simulated heats of adsorption for CO_2_ at 298 K.

In order to better understand the CO_2_ adsorption behaviour in FMOF-1 at higher pressure (see Fig. 1), we also simulated CO_2_ adsorption isotherms at 298 K up to 50 bar for different FMOF-1a–c structures and compared the results to the simulated uptakes obtained for FMOF-2 and the experimental isotherm (Fig. 9a). The FMOF-2 structure is obtained from annealing FMOF-1 and recrystallizing from a toluene/acetonitrile solution and consists of two enlarged types of pores: hexagonal channels that are *ca.* 18 Å in width and triangular cages that are *ca.* 10 Å in diameter.^46^ The maximum amount adsorbed for CO_2_ at 298 K is 3.2 mol kg^–1^ or 23.4 molecules per unit cell in FMOF-1c. This value is in excellent agreement with the result from neutron powder diffraction experiments at 290 K, where CO_2_ uptake capacity of 3.3 mol kg^–1^ (24 molecules per unit cell) can be obtained when all three CO_2_ adsorption sites in the large cavity are fully occupied under 61 bar of CO_2_ loading (see Fig. 5). However, with a small increase in temperature, at 298 K, the simulated CO_2_ uptake is significantly lower than the experimental uptake even in the expanded FMOF-1b structure, suggesting further enlargement of the structure at this temperature. It is very clear from Fig. S2† that the CO_2_ adsorption of FMOF-1 at room temperature represents a marked deviation from the typical type 1 isotherm. Generally, Toth fit modeling is carried out on type 1 isotherms. In order to obtain a good Toth fit for the CO_2_ adsorption at room temperature, the pressure range is scaled down so as to compare experimental data with the simulation data. Fig. S10† shows that the Toth fit agrees well with the FMOF-1b structure. Indeed, the simulated saturation loading of CO_2_ in FMOF-2 shows better agreement with the experimental data at elevated pressures around 40 bar, suggesting expansion of FMOF-1 to a more expanded polymorph at higher pressures up to 60 bar. The collective experience of the Omary group with such M-Tz(R_F_)_2_ compositions suggests the prevalence of polymorphism and crystallographic isomerism; this is so both with and without gas adsorption assistance. As such, the presumed significant expansions (or compressions) suggested by the closer proximity of the experimental high-pressure CO_2_ uptake in FMOF-1 at ambient temperature to the simulated data for the porous-most “emptied” N_2_@FMOF-1 adsorption adduct at 90 K or – better yet – the FMOF-2 structure at 100 K should not be surprising for this highly flexible material.

Fig. 9b compares the isosteric heats of adsorption (*Q*_st_) obtained from GCMC simulations to the values obtained from variable-temperature experiments. The predicted *Q*_st_ values were obtained from the fluctuations of the potential energy over the production cycles in the GCMC simulations for each pressure point. The experimental heats of adsorption were obtained using the Clausius–Clapeyron equation on isotherms from several temperatures (see ESI† for details). In reasonably good agreement with the experimental data, the simulated *Q*_st_ values for the three FMOF-1 structures rise smoothly from ∼16 to 25 kJ mol^–1^ as the loading increases due to an increasing contribution from attractive CO_2_–CO_2_ interactions (Fig. S11†). The adsorption heats for FMOF-2 are higher (∼22 kJ mol^–1^) at low loadings due to strong adsorption of CO_2_ in the small triangular pores. In general, these moderate adsorption heats lie in the typical range for CO_2_ adsorption in MOFs.^61,62^ In Fig. S11,† the average adsorbate–adsorbent and adsorbate–adsorbate energies from GCMC simulations are shown for CO_2_ adsorption in FMOF-1c at 298 K as a function of pressure. The van der Waals interactions between CO_2_ and FMOF-1 are clearly dominant with ∼–12 kJ mol^–1^ contribution, and the coulombic components of the total interaction energy are very small, ∼–2 kJ mol^–1^. These results suggest that the fluorine-lined cylindrical pore creates an electrostatic potential that is not very favourable for CO_2_ adsorption.

**Fig. 10:**
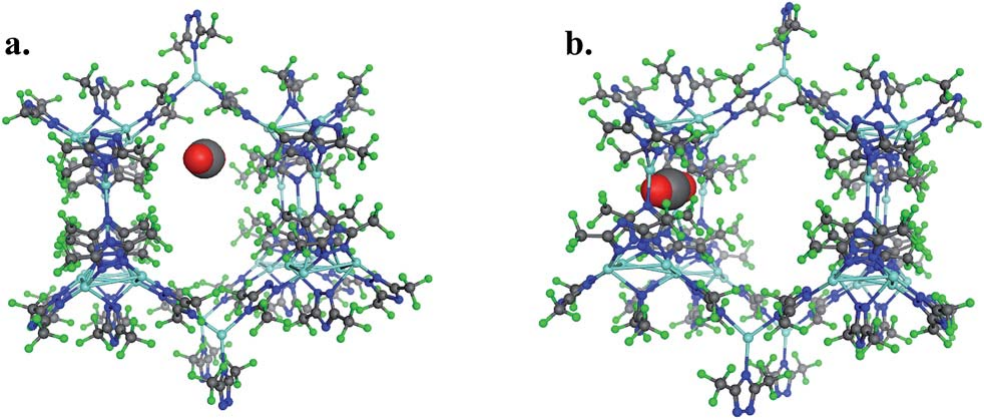
(a) CO_2_ in the cylindrical channel within the extended FMOF-1a structure and (b) CO_2_ in the small cavity within the extended FMOF-1b structure.

To provide another perspective on guest molecule interactions with FMOF-1, QM methods (UDFT with dispersion) employing cluster models were used to determine the free energy of binding for several guest molecules. The binding sites used in these calculations are located in the cylindrical channel and the small cavity. These sites are represented in Fig. 10 in the extended MOF structure with an overlay of the guest geometry from the cluster calculations.

**Fig. 11:**
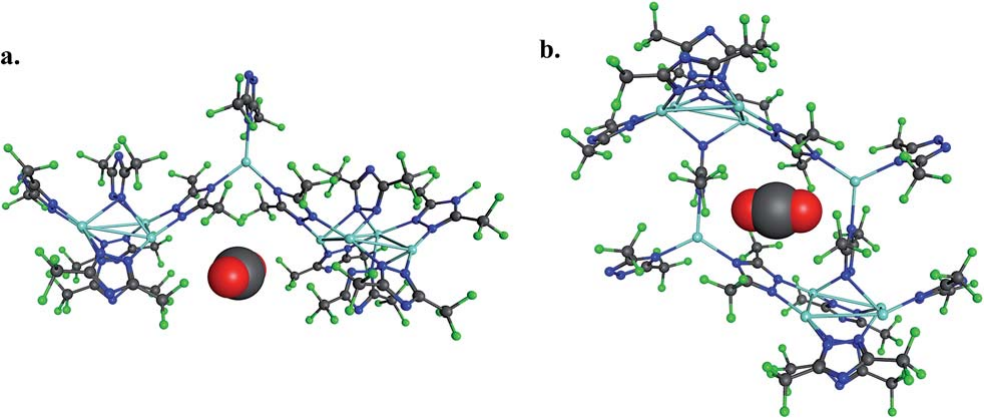
(a) CO_2_ interaction with the 184-atom cluster model for the cylindrical channel taken from the FMOF-1a structure. (b) CO_2_ interaction with the 194-atom cluster model for the small cavity taken from the FMOF-1b structure.

The 184-atom extended model for the cylindrical channel binding site is taken from the FMOF-1a structure and shown in Fig. 11a. The extended model of the cylindrical channel with coordinated Ag and Tz was truncated with complete metal coordination spheres and ligands. By preserving the metal to ligand ratio, no atom substitutions were needed. The calculated binding free energies of CO_2_, N_2_, and H_2_ in the cylindrical channel are given in Table 1. The binding free energies follow the order CO_2_ > N_2_ > H_2_ in the cylindrical channel, indicating selective adsorption of CO_2_. The dispersion contribution increases the magnitude of the binding free energy by ∼59–66% in these calculations. Similar results with respect to the relative contribution of dispersion were found by Neaton for carbon dioxide binding in Mg–MOF-74.^44^

**Table 1:** DFT binding free energies of various guest molecules with FMOF-1a cylindrical channel and FMOF-1b small cavity

Guest	Cylindrical channel		Small cavity	
	PBE0 (kJ mol^–1^)	PBE0-D3 (kJ mol^–1^)	PBE0 (kJ mol^–1^)	PBE0-D3 (kJ mol^–1^)
CO_2_	–9.2	–23.2	138.6	94.5
N_2_	–7.9	–19.3	–9.0	–39.6
H_2_	–2.1	–6.6	–3.5	–16.8

The binding free energies in the small cavity of FMOF-1b were also determined for CO_2_, N_2_, and H_2_ using the truncated model shown in Fig. 11b. The small cavity shows stronger binding for N_2_ and H_2_ than in the cylindrical channel, with a similar dispersion correction making up 62–79% of the total binding free energy. The binding of CO_2_ in the truncated model of the small cavity is not energetically favourable, in agreement with GCMC simulations and neutron diffraction experimental results.

### Effect of humidity on CO_2_ uptake

**Fig. 12:**
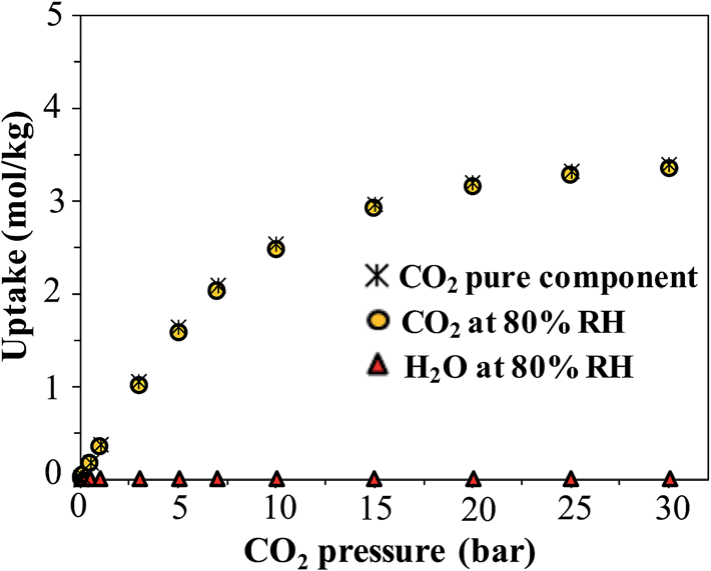
Simulated excess adsorption isotherms at 298 K for pure component CO_2_ and a mixture of CO_2_ at different pressures with water at 80% relative humidity in FMOF-1c.

Finally, we consider the effect of humidity on the CO_2_ uptake of FMOF-1. Water is an ever-present component of flue gas, and we hypothesized that hydrophobic MOFs like FMOF-1 should show negligible loss of CO_2_ capacity in the presence of water vapor. To test this, we carried out GCMC simulations of CO_2_ adsorption in humid conditions. Fig. 12 depicts the excess amount adsorbed for a mixture of CO_2_ and H_2_O in FMOF-1c at 80% relative humidity and at different CO_2_ pressures. Although the CO_2_ uptake at 0.15 bar (the relevant condition in flue gas)^63^ is only *ca.* 0.05 mol kg^–1^ (0.3 molecules per unit cell), it is shown that for such a superhydrophobic MOF, the uptake of CO_2_ is not influenced by the presence of water vapor at all. There is essentially no co-adsorption of water in the presence of CO_2_, thereby explaining why the CO_2_ adsorption amount is similar under dry or humid conditions. This result supports the hypothesis that FMOFs can be promising to diminish the effect of humidity on CO_2_ adsorption performance.

## Conclusions

We combined atomically detailed calculations and experiments to provide insight into the adsorption of CO_2_ and other molecules in FMOF-1. First, the force field model for FMOF-1 was verified by comparing simulated adsorption isotherms for a variety of adsorbates in FMOF-1 with existing experimental data. Adsorption isotherms of N_2_ and O_2_ support previous suggestions that FMOF-1 undergoes major structural changes in the presence of guest molecules. This flexibility, however, has little effect on the loading of larger adsorbates such as *n*-hexane and benzene. The two-step isotherms observed for both N_2_ and O_2_ can be explained by analyzing simulated adsorption isotherms in both contracted and expanded FMOF-1 structures and the accessibility of the small pockets to these smaller adsorbates. We measured CO_2_ adsorption isotherms in FMOF-1 and found good agreement between experiment and simulations using the FMOF-1c structure at sub-ambient temperatures. Simulations of CO_2_ adsorption in FMOF-1 along with neutron powder diffraction measurements show that CO_2_ molecules cannot fit in the small pores of FMOF-1 and reside in the large channels at three distinct adsorption sites. The experimentally measured CO_2_ isotherms near and above room temperature suggest significant framework expansion at high CO_2_ pressures. Contact angle measurements confirm FMOF-1's previously reported hydrophobic nature, and Monte Carlo simulations predict no uptake of water even up to the vapor pressure of water. Simulations of CO_2_ adsorption in the presence of 80% relative humidity show that the amount of CO_2_ adsorbed is essentially the same as in the absence of humidity, validating our hypothesis that hydrophobic MOFs could hold promise for CO_2_ capture from flue gas.

## Conflict of interest

R. Q. S. has a financial interest in the start-up company NuMat Technologies, which is seeking to commercialize metal–organic frameworks.
