# Supplementary material for: Adsorption and molecular siting of CO2, water, and other gases in the superhydrophobic, flexible pores of FMOF-1 from experiment and simulation
Source: Chem Sci. 2017 Mar 10;8(5):3989–4000. doi: 10.1039/c7sc00278e (PMC5433493; doi:10.1039/c7sc00278e)
Supplement: Supplementary file 1 [file SC-008-C7SC00278E-s001.pdf]

Electronic Supplementary Material (ESI) for Chemical Science.
This journal is © The Royal Society of Chemistry 2017

Electronic Supplementary Information (ESI)

Adsorption and Molecular Siting of CO_2, Water, and Other Gases in the Superhydrophobic, Flexible Pores of FMOF-1 from Experiment and Simulation

Peyman Z. Moghadam,[†,$] Joshua F. Ivy,[‡,$] Ravi K. Arvapally,[‡,$] Antonio M. dos Santos,[§] John C. Pearson,[‡,▿] Li Zhang,[†,⊥] Emmanouil Tylianakis,[||] Pritha Ghosh,[†] Iain W. H. Oswald,[‡] Ushasree Kaipa,[‡] Xiaoping Wang,[§]* Angela K. Wilson,[‡ ▿]* Randall Q. Snurr,[†]* and Mohammad A. Omary[‡]*

[†]*Department of Chemical & Biological Engineering, Northwestern University, 2145 Sheridan Road, Evanston, IL 60208 − 3120, USA*

[‡]*Department of Chemistry, University of North Texas, Denton, Texas 76203, USA*

[§]*Neutron Sciences Directorate, Oak Ridge National Laboratory, Oak Ridge, TN 37831, USA*

[⊥]*Department of Chemistry, Zhejiang Sci-Tech University, Hangzhou, China*

[||]*Department of Materials Science & Technology, University of Crete, Voutes Campus, Heraklion, Crete GR-71003, Greece*

[▿]*Department of Chemistry, Michigan State University, East Lansing, MI 48824-1322, USA*

[$]*Authors with equal contribution (co-1st authors)*

*E-mail: wangx@ornl.gov

*E-mail: wilson@chemistry.msu.edu

*E-mail: snurr@northwestern.edu

*E-mail: omary@unt.edu

CO_2 isotherms at 298 K up to 55 bar

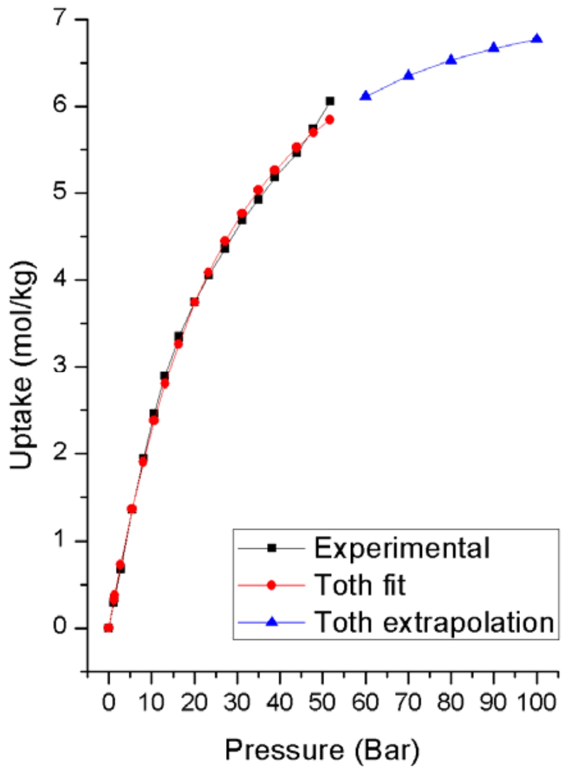

Figure S1. CO_2 adsorption isotherm of a powder sample of FMOF-1 at 298 K, including the experimental data (black) and the fitting to the Toth equation within the experimental range (red) and beyond (blue).

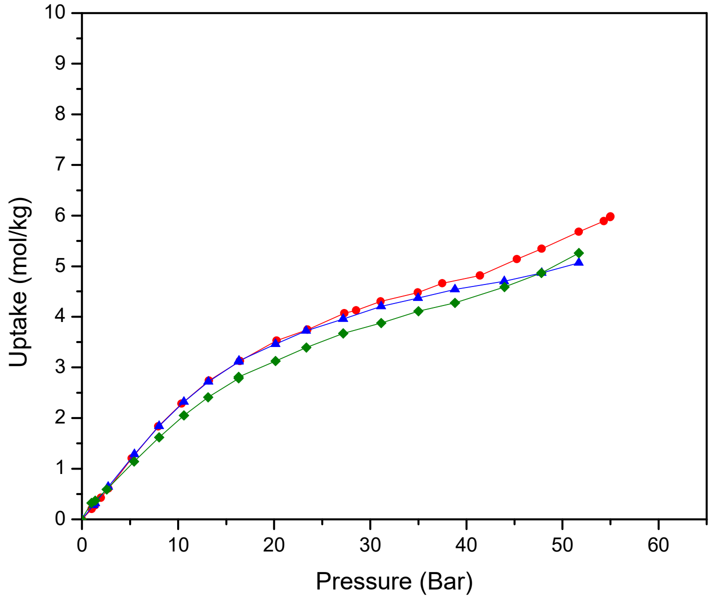

Figure S2. Representative reproduction attempts for the CO_2 adsorption isotherms at 298 K up to 55 bar using different samples from multiple batches of activated powders of FMOF-1.

Isosteric Heats of Adsorption Calculation

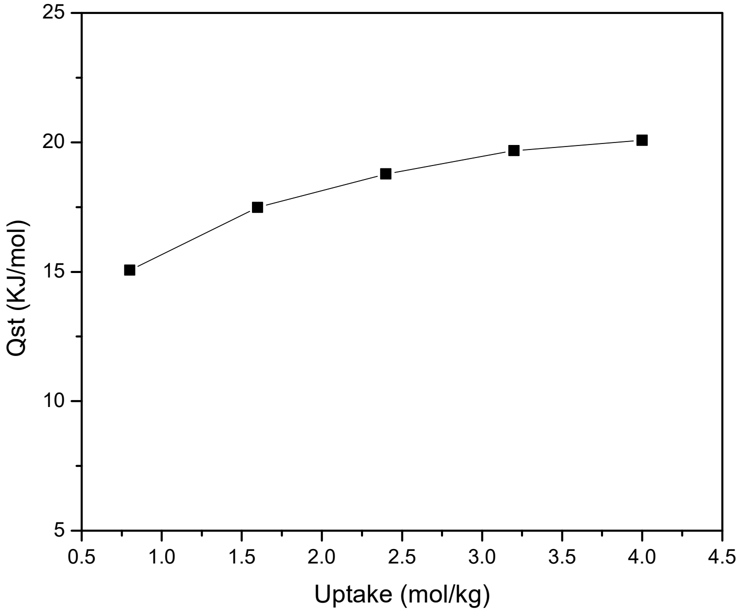

Figure S3. Isosteric heat of adsorption of FMOF-1 determined from experimental data.

Table S1. Neutron powder diffraction experimental details for **FMOF-1c + CO_2**.

Crystal Data	
Chemical formula	$C_{12.625}$ Ag_3 F_{18} N_9 $O_{1.25}$
M_r	963.28
Crystal system, space group	Tetragonal, *I-42d*
Temperature (K)	290 (2)
Pressure (bar)	61 (1)
a, *c* (Å)	13.9713 (7), 37.713 (4)
V (Å^3)	7361.4 (7)
Z	8
Radiation type	Spallation neutron
Data collection	
Diffractometer	SNS SNAP http://neutrons.ornl.gov/snap
Specimen mounting	6 mm diameter vanadium cup in a cylindrical TiZr Pressure cell
Data collection mode	Transmission
Scan method	Neutron time of flight powder diffraction
Refinement	
R factors and goodness of fit	R_p = 0.015, R_{wp} = 0.016, R_{exp} = 0.009, $R(F)$ = 0.145, $R(F^2)$ = 0.178, χ^2 = 3.512
No. of data points	1661
No. of parameters	14

Computer programs: GSAS-II (Toby & Von Dreele, J. Appl. Cryst. 2013, 46, 544-549); Structure refined with rigid body constraints.

Evolution of the neutron diffraction patterns of FMOF-1 with CO_2

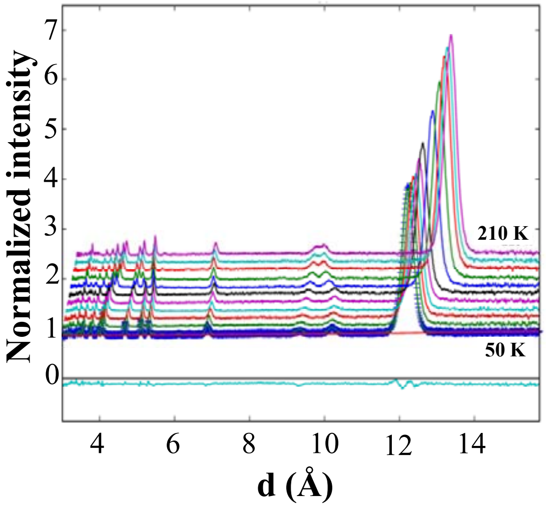

Figure S4. Evolution of neutron powder pattern of FMOF-1 with absorbed CO_2 in temperature range 50 K to 210 K. The solid line (cyan) is the residuals from the observed and refined neutron diffraction profiles of the FMOF-1 sample with adsorbed CO_2 at 50 K. wR = 1.67%,GOF = 3.81, N_{obs} = 1659, N_{vals} = 17. Space group I-42d, a = 12.7855 (6) Å, c = 40.755(4) Å, V = 6662.2(5) Å^3.

Geometric characterization of FMOF-1 structures

The pore size distributions and geometric characterization for FMOF-1a-c structures are shown in Figure S5 and Table S2, respectively. The window and pore diameters were obtained using the method described by Sarkisov and Harrison.[1] Other geometric properties such as the accessible surface area, void fraction and pore volume were calculated using methods reported by Düren et al.[2] and Frost et al.[3].

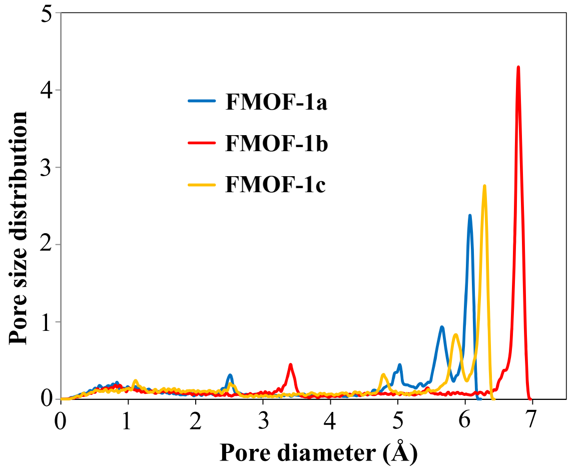

Figure S5. Pore size distributions for FMOF-1 structures. The pore sizes are calculated using the method described by Gelb and Gubbins.[4]

Table S2. Geometric properties for the FMOF-1 structures studied in this work

Structure	Surface area	He Void Fraction	Channel diam.	Channel window diam.	Small cavity diam.	Small cavity window diam.
	m^2/g	_	Å	Å	Å	Å
**FMOF-1a**	777	0.40	6.1	5.0	2.5	0.5-1.0
**FMOF-1b**	870	0.45	6.8	6.0	3.5	2.5-3.0
**FMOF-1c**	782	0.41	6.3	5.5	2.5	0.5-1.0

FMOF-1 model

The LJ parameters for FMOF-1 atoms were taken from the Universal Force Field (UFF).[5] The partial charges on CF_3 groups were taken from the work of Dalvi et al.[6] Partial charges for C and F atoms in CF_3 group are +0.51 and -0.17, respectively. The partial charges for all other atoms in FMOF-1 were obtained from DFT calculations using the B3LYP functional and the 6-31+G* basis set in Gaussian 09[7]. Partial atomic charges were extracted using the ChelpG method.[8] Table S3 shows the LJ parameters for all atom types in FMOF-1.

Table S3. LJ parameters for FMOF-1 atoms

Atom type	ε/k_B (K)	σ(Å)	Force field
N	34.6	3.26	UFF[5]
C	52.4	3.43	UFF[5]
F	25.2	3.09	UFF[5]
Ag	18.116	2.806	UFF[5]

Adsorbate models

The TraPPE force field was used to model all adsorbates (N_2[9], O_2[10], CO_2[9], hexane[11] and benzene[12]) except for water, which was described with the TIP4P model[13]. The LJ parameters along with partial charges for all adsorbates are listed in Table S4.

Table S4. LJ parameters and partial charges for all adsorbates studied in this work

Adsorbate	Atom type	ε/k_B (K)	σ(Å)	q(e)	Force field
**Oxygen**	O_O_2	49	3.02	-0.113	TraPPE[10]
	COM_O_2	-	-	0.226	TraPPE[10]
**Nitrogen**	N_N_2	36	3.31	-0.482	TraPPE[9]
	COM_N_2	-	-	0.964	TraPPE[9]
**Hexane**	CH_3	98.0	3.75	-	TraPPE[11]
	CH_2	46.0	3.95	-	TraPPE[11]
**Carbon dioxide**	O_CO_2	79.0	3.05	-0.35	TraPPE[9]
	C_CO_2	27.0	2.80	0.70	TraPPE[9]
**Benzene**	C_benz	30.7	3.6	-0.09	TraPPE[12]
	H_benz	25.5	2.36	0.09	TraPPE[12]
**Water**	Ow_TIP4P	78.0	3.15	-	TIP4P[13]
	H_TIP4P	-	-	0.52	TIP4P[13]
	M_TIP4P	-	-	-1.04	TIP4P[13]

Additional simulation results

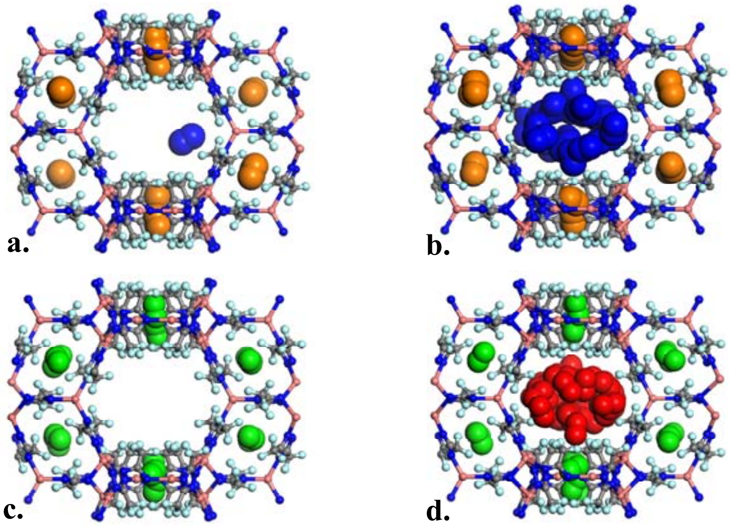

Figure S6 . GCMC simulation snapshots showing sequential pore filling of small pockets and large channels for N_2 and O_2 in FMOF-1b at 77 K. a) N_2 at 1.58 Pa b) N_2 at 99990 Pa c) O_2 at 0.1 Pa and d) O_2 at 20000 Pa at 77 K. Nitrogen molecules adsorbed in the large channels and small pockets are illustrated with blue and orange, respectively. Oxygen molecules adsorbed in large channels and small pockets are illustrated with red and green, respectively.

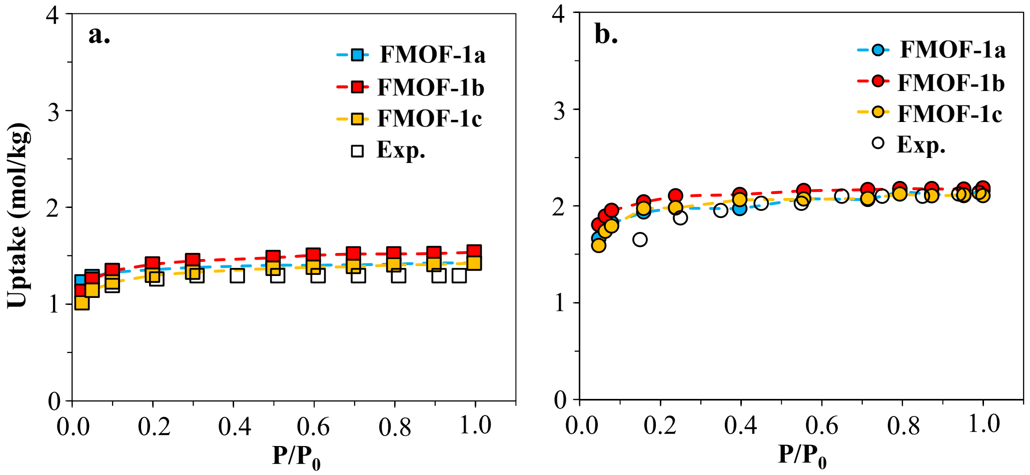

Figure S7. Simulated and experimental[14] adsorption isotherms for a) hexane and b) benzene in all FMOF-1 structures at 298 K. P_0 is the experimental saturation pressure of each adsorbate.

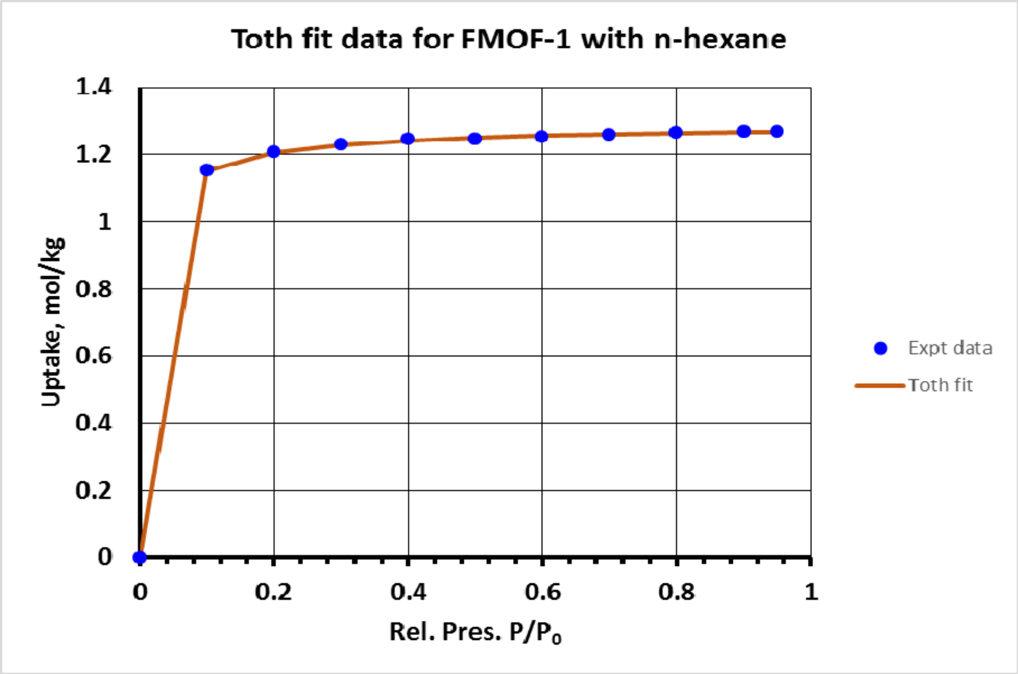

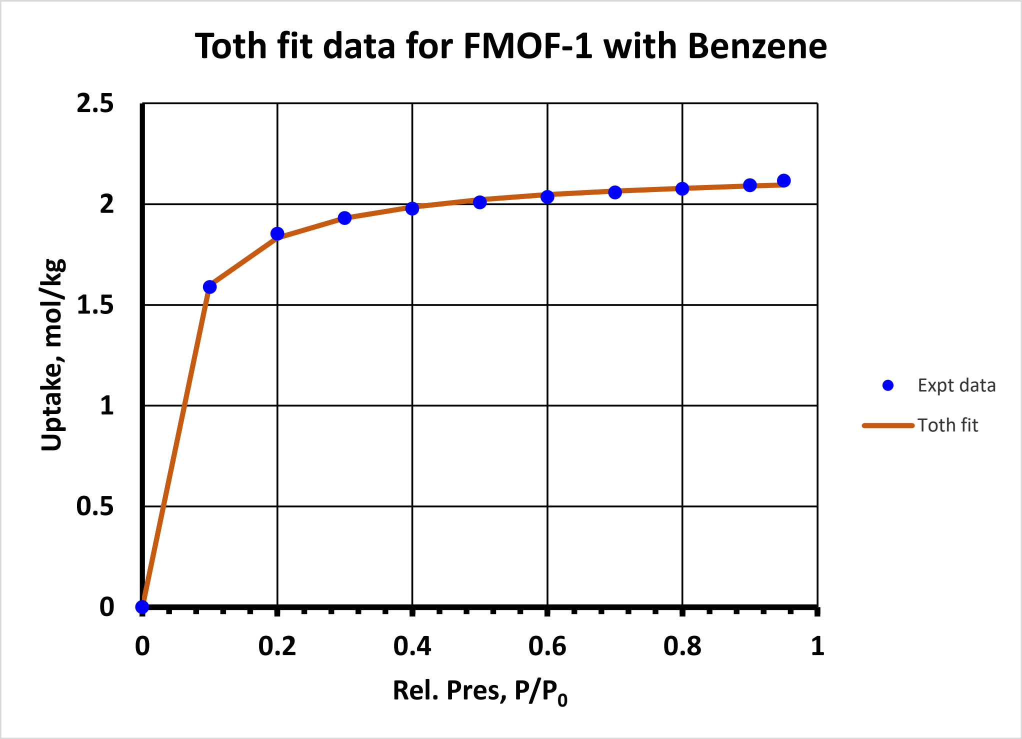

Figure S8. Toth fitting of experimental[14] adsorption isotherms for a/top) *n*-hexane and b/bottom) benzene for FMOF-1 at 298 K. P_0 is the experimental saturation pressure of each adsorbate.

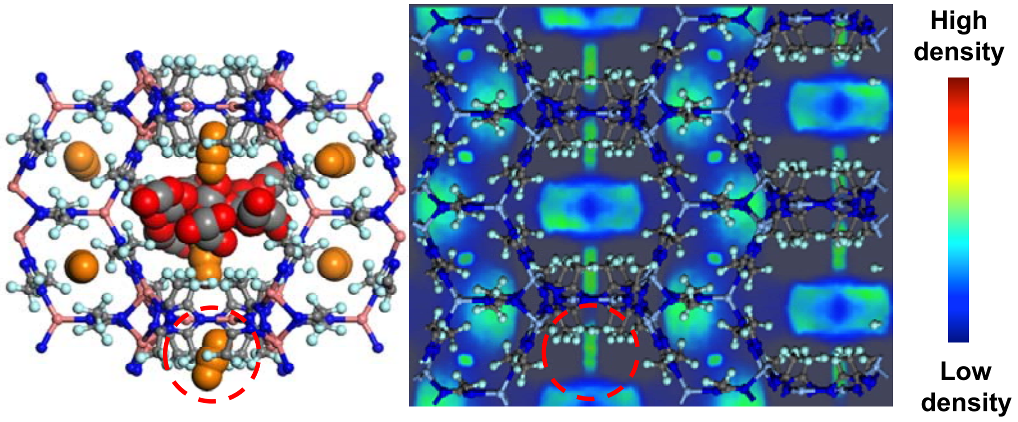

Figure S9. GCMC simulation snapshot (left) and density profiles (right) for CO_2 adsorption in FMOF-1b at saturation loading and 278 K. The CO_2 presence at the entrance of the small pockets is highlighted by the dashed red circle.

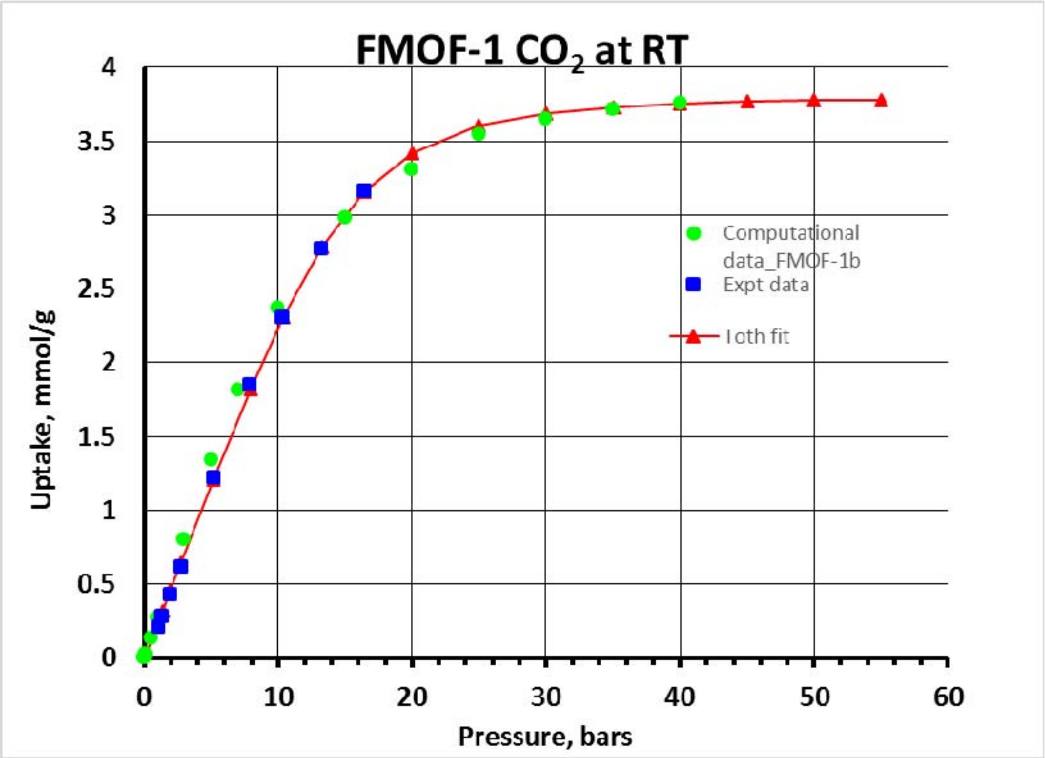

Figure S10. Comparison of the experimental, computation, and Toth isotherms of CO_2 adsorption in FMOF-1 at room temperature (RT).

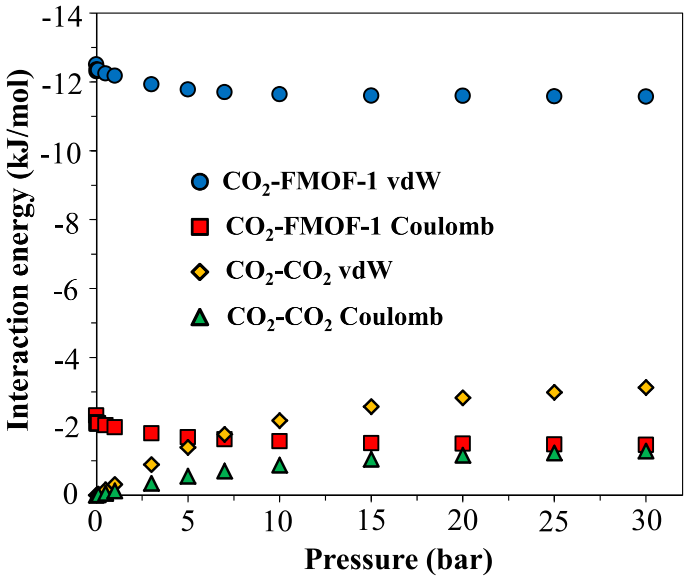

Figure S11. The breakdown of potential energy into adsorbate-adsorbate and adsorbate-adsorbent contributions for CO_2 adsorption in FMOF-1c obtained from GCMC simulations at 278 K.

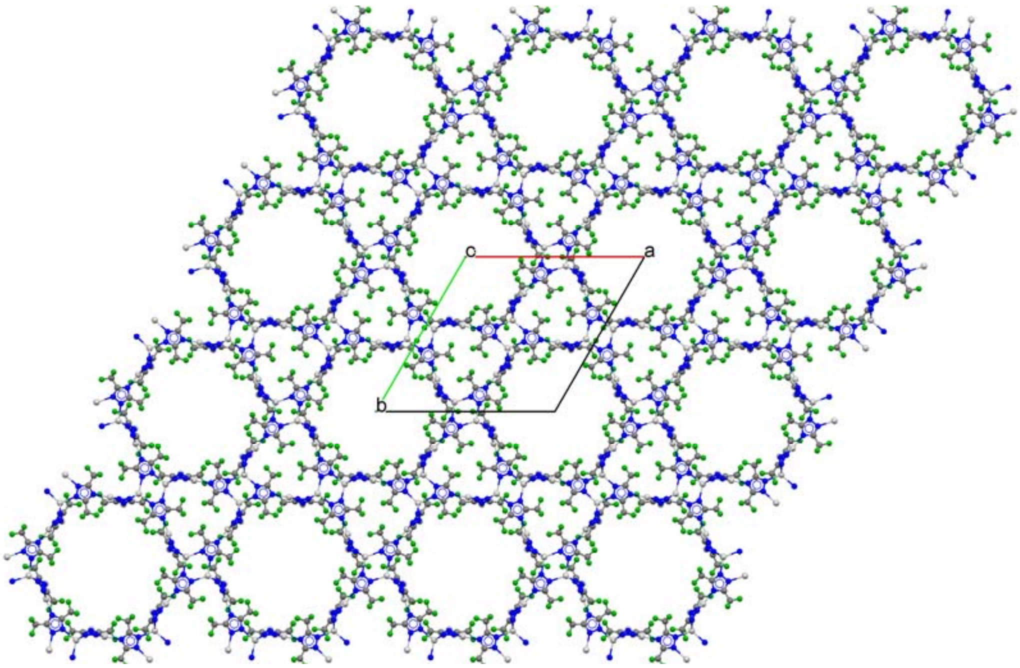

Figure S12. The backbone of the FMOF-2 guest-free framework, adapted from the published structure of the toluene adsorption adduct published earlier (see ref. 14(b) below).

ESI References

1. Sarkisov, L.; Harrison, A.; Sarkisov, L.; Harrison, A., Computational structure characterisation tools in application to ordered and disordered porous materials. *Molecular simulation* **2011,** *37* (15), 1248-1257.

2. Duren, T.; Bae, Y.-S.; Snurr, R. Q., Using molecular simulation to characterise metal-organic frameworks for adsorption applications. *Chemical Society Reviews* **2009,** *38* (5), 1237-1247.

3. Frost, H.; Düren, T.; Snurr, R. Q., Effects of Surface Area, Free Volume, and Heat of Adsorption on Hydrogen Uptake in Metal−Organic Frameworks. *The Journal of Physical Chemistry B* **2006,** *110* (19), 9565-9570.

4. Gelb, L. D.; Gubbins, K. E., Pore Size Distributions in Porous Glasses: A Computer Simulation Study. *Langmuir* **1999,** *15* (2), 305-308.

5. Rappe, A. K.; Casewit, C. J.; Colwell, K. S.; Goddard, W. A.; Skiff, W. M., UFF, a full periodic table force field for molecular mechanics and molecular dynamics simulations. *Journal of the American Chemical Society* **1992,** *114* (25), 10024-10035.

6. Dalvi, V. H.; Srinivasan, V.; Rossky, P. J., Understanding the Effectiveness of Fluorocarbon Ligands in Dispersing Nanoparticles in Supercritical Carbon Dioxide. *The Journal of Physical Chemistry C* **2010,** *114* (37), 15553-15561.

7. Frisch, M. J.; Trucks, G. W.; Schlegel, H. B.; Scuseria, G. E.; Robb, M. A.; Cheeseman, J. R.; Scalmani, G.; Barone, V.; Mennucci, B.; Petersson, G. A.; Nakatsuji, H.; Caricato, M.; Li, X.; Hratchian, H. P.; Izmaylov, A. F.; Bloino, J.; Zheng, G.; Sonnenberg, J. L.; Hada, M.; Ehara, M.; Toyota, K.; Fukuda, R.; Hasegawa, J.; Ishida, M.; Nakajima, T.; Honda, Y.; Kitao, O.; Nakai, H.; Vreven, T.; Montgomery Jr., J. A.; Peralta, J. E.; Ogliaro, F.; Bearpark, M. J.; Heyd, J.; Brothers, E. N.; Kudin, K. N.; Staroverov, V. N.; Kobayashi, R.; Normand, J.; Raghavachari, K.; Rendell, A. P.; Burant, J. C.; Iyengar, S. S.; Tomasi, J.; Cossi, M.; Rega, N.; Millam, N. J.; Klene, M.; Knox, J. E.; Cross, J. B.; Bakken, V.; Adamo, C.; Jaramillo, J.; Gomperts, R.; Stratmann, R. E.; Yazyev, O.; Austin, A. J.; Cammi, R.; Pomelli, C.; Ochterski, J. W.; Martin, R. L.; Morokuma, K.; Zakrzewski, V. G.; Voth, G. A.; Salvador, P.; Dannenberg, J. J.; Dapprich, S.; Daniels, A. D.; Farkas, Ö.; Foresman, J. B.; Ortiz, J. V.; Cioslowski, J.; Fox, D. J. *Gaussian 09*, Gaussian, Inc.: Wallingford, CT, USA, 2009.

8. Breneman, C. M.; Wiberg, K. B., Determining atom-centered monopoles from molecular electrostatic potentials. The need for high sampling density in formamide conformational analysis. *Journal of Computational Chemistry* **1990,** *11* (3), 361-373.

9. Potoff, J. J.; Siepmann, J. I., Vapor–liquid equilibria of mixtures containing alkanes, carbon dioxide, and nitrogen. *AIChE Journal* **2001,** *47* (7), 1676-1682.

10. Zhang, L.; Siepmann, J. I., Direct calculation of Henry's law constants from Gibbs ensemble Monte Carlo simulations: nitrogen, oxygen, carbon dioxide and methane in ethanol. *Theor Chem Acc* **2006,** *115* (5), 391-397.

11. Martin, M. G.; Siepmann, J. I., Transferable Potentials for Phase Equilibria. 1. United-Atom Description of n-Alkanes. *The Journal of Physical Chemistry B* **1998,** *102* (14), 2569-2577.

12. Rai, N.; Siepmann, J. I., Transferable Potentials for Phase Equilibria. 9. Explicit Hydrogen Description of Benzene and Five-Membered and Six-Membered Heterocyclic Aromatic Compounds. *The Journal of Physical Chemistry B* **2007,** *111* (36), 10790-10799.
13. Jorgensen, W. L.; Chandrasekhar, J.; Madura, J. D.; Impey, R. W.; Klein, M. L., Comparison of simple potential functions for simulating liquid water. *The Journal of Chemical Physics* **1983,** *79* (2), 926-935.
14. (a) Yang, C.; Wang, X.; Omary, M. A., Fluorous Metal−Organic Frameworks for High-Density Gas Adsorption. *Journal of the American Chemical Society* **2007,** *129* (50), 15454-15455; (b) Yang, C.; Kaipa, U.; Mather, Q. Z.; Wang, X.; Nesterov, V.; Venero, A. F.; Omary, M. A., Fluorous Metal–Organic Frameworks with Superior Adsorption and Hydrophobic Properties toward Oil Spill Cleanup and Hydrocarbon Storage. *Journal of the American Chemical Society* **2011,** *133* (45), 18094-18097.
